# Clinical effectiveness of supraspinatus tendon reconstruction using autogenous fascia latas for irreparable posterosuperior massive rotator cuff tears: study protocol for a randomized, controlled clinical trial

**DOI:** 10.1186/s13063-023-07741-y

**Published:** 2023-10-31

**Authors:** Lin Ma, Xiaoli Gou, Binghua Zhou

**Affiliations:** Department of Sports Medicine, Southwest Hospital, Army Medical University, No. 30 Gaotanyan Main Street, Chongqing, 400038 China

**Keywords:** Rotator cuff injuries, Reconstruction, Fascia latas, Massive rotator cuff tears, Randomized, Controlled clinic trial

## Abstract

**Background:**

Supraspinatus tendon reconstruction (STR) was recently introduced as a new treatment option for irreparable posterosuperior massive rotator cuff tears (IPMRCT). STR was thought to be more advantageous than superior capsule reconstruction (SCR) for restoring supraspinatus (SSP) dynamics. However, there has been no prospective randomized controlled study on the early clinical efficacy of STR.

**Methods:**

A single-site, prospective, observers and patients double-blinding randomized controlled trial was designed. Fifty-eight patients aged 50–85 years with IPMRCT will be randomized 1:1 to receive either STR or SCR. The clinical outcomes were evaluated using the American Society for Shoulder and Elbow Surgery (ASES) score, range of motion (ROM), visual analogue scale (VAS) for pain, acromiohumeral distance (AHD), Goutlliar grade for fatty infiltration in the SSP, Sugaya grade for the autogenous fascia latas, isokinetic muscle strength testing and surface electromyography (EMG) testing for shoulder abduction muscle strength and complications.

**Discussion:**

The results of this study will contribute to the treatment algorithm of IPMRCT and assist surgeons in making treatment decisions. This is the first randomized controlled trial to compare the effects of STR and SCR for the treatment of IPMRCT.

**Trial registration:**

We registered the trial in chictr.org.cn on July 17, 2023 (register number: ChiCTR2300073716). Items from the WHO trial registry were found within the protocol.

## Administrative information

Note: The numbers in curly brackets in this protocol correspond with SPIRIT checklist item numbers. The items were grouped based on similarity (see http://www.equator-network.org/reporting-guidelines/spirit-2013-statement-defining-standard-protocol-items-for-clinical-trials/).

**Table:** 

Title {1}	Clinical effectiveness of supraspinatus tendon reconstruction using autogenous fascia latas for irreparable posterosuperior massive rotator cuff tears: study protocol for a prospective randomized, controlled clinical trial
Trial registration {2a and 2b}.	ChiCTR, ChiCTR2300073716. Registered 17 July 2023,https://www.chictr.org.cn/bin/project/edit?pid=183658
Protocol version {3}	Version 1.3 (20/04/2023)
Funding {4}	Chongqing Yingcai Projects for Creative Leading Talents (CQYC20200303135).
Author details {5a}	Department of sports medicine, Southwest Hospital, Army Medical University, No. 30 Gaotanyan Main Street, Chongqing, China 400038
Name and contact information for the trial sponsor {5b}	Chongqing Health Commission, Huang Ke, + 86 19923516611
Role of sponsor {5c}	The study sponsor had no direct role in the study design. nor the collection, management, analysis or interpretation of the data. They will have no role in the writing of associated publications or the decision to submit papers for publication. This research programme will be fund by Chongqing Yingcai Projects for Creative Leading Talents (CQYC20200303135).

## Introduction

### Background and rationale {6a}

IPMRCT results in severe shoulder dysfunction and shoulder instability. SCR and bridging patches have been increasingly used to treat IPMRCT [1, 2]. In the SCR technique, the patch graft is fixed on the glenoid in the medial direction and on the footprint of the rotator cuff in the lateral direction. SCR technique was introduced as an innovative treatment for MRCT in 2013 [1] and achieved a long-term successful outcome [2]. SCR restored superior glenohumeral joint stability and then improved shoulder joint function [1, 3]. Retear rates were seen with partial cuff repairs (45%), graft interposition (21%) and SCR (21%), respectively [3]. As a whole, SCR was a validated, safe option compared with partial repair or simply physiotherapy. SCR was an inexpensive option too compared with reverse shoulder arthroplasty. However, SCR did not restore the anatomy or dynamic function of the SSP tendon. Bridging techniques with different patches that mimic the anatomy of the SSP tendon have been reported [2, 4]. However, this technique should be used carefully because of the high retear rate associated with the patch graft-tendon interface, the poor mechanical properties of allogenic grafts and the possibility of inflammation [4]. The STR technique using the autogenous fascia latas (FL) was recently introduced to treat IPMRCT [5]. The technique achieved a stable anatomic and dynamic reconstruction of the SSP tendon with fascia-muscle fusion. It was hypothesized that STR would have better clinical outcomes than SCR. However, there are still no reports of the clinical outcomes.

The intention of this study was to evaluate the clinical efficacy of STR for the treatment of IPMRCT in a prospective randomized controlled study to provide evidence-based medical evidence for its extensive development.

### Objectives {7}

The objectives of this trial were to compare the clinical effects of STR and SCR using autogenous fascia latas for the repair of IPMRCT. We hypothesized that STR would have a better clinical outcome than SCR.

### Trial design {8}

**Fig. 1:**
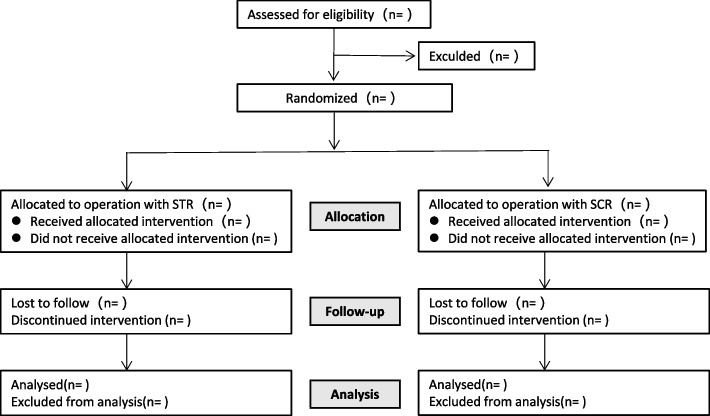
CONSORT flow diagram of the progress of the study

The trial is designed as a 1:1 randomized, controlled, observers and patients double-blinded single-centre trial with two parallel groups, which will compare the STR versus SCR using autogenous fascia latas for the repair of IPMRCT. The enrolled patients were randomized in a 1:1 ratio into two treatment groups (STR group and SCR group) by a simple computer-generated randomization system (https://castoredc.com). Double-blinding was used, in which patients and evaluators were blinded to minimize ascertainment bias. It was registered in the Chinese Clinical Trial Registry (ChiCTR.org ID: ChiCTR2300073716). The study protocol was approved by our institutional review board, and all subjects will be asked to finish a written informed consent. The flow chart of the inclusion and exclusion of trial participants is given in Fig. 1.

## Methods: participants, interventions and outcomes

### Study setting {9}

The study will be conducted in the First Affiliate Hospital of Army Medical University China from Sep 2023 to August 2028. Patients with IPMRCT requiring arthroscopic surgeries will be transferred from our outpatient centre. After confirming the patient’s eligibility for inclusion and completing a baseline-level assessment, the investigator will give a detailed presentation of the study protocol and potential risks and answer all the questions raised by the patients. Each patient will sign an informed consent form. Upon enrolment, participants will be coded with a unique number.

### Eligibility criteria {10}

#### Inclusion criteria

1. Age 50–85 years old.
2. Massive rotator cuff tear identified by preoperative MRI imaging technology (sagittal anterior and posterior tear length ≥ 5 cm or involving two or more rotator cuff tendon tears).
3. The intraoperative evidence was IPMRCT (standard for irreparable rotator cuff injury: after full release, tissue forceps were used to test the tension of the broken rotator cuff stump, and it was found that the rotator cuff stump could not effectively cover the footprint under moderate pulling tension).
4. Preoperative imaging examination Hamada classification within type 3.
5. Agree to undergo arthroscopic STR or SCR operation.

#### Exclusion criteria

1. There is a previous fracture or nerve injury around the shoulder joint.
2. Suffered cerebral infarction or cerebral haemorrhage in the past 6 months.
3. Experienced unstable angina pectoris or myocardial infarction in the past 6 months.
4. Psychiatric problems that precluded informed consent or inability to read or write.
5. Preoperative infection.
6. Complicated with other serious medical diseases.

### Who will take informed consent? {26a}

Patients with IPMRCT who met the inclusion will choose whether to join the clinical trial in the outpatient department. Dr. Zhou will inform the patients with IPMRCT that they have met the inclusion criteria for the clinical trial. After confirming the patient’s eligibility for inclusion and completing a baseline-level assessment, the main investigator will give a detailed presentation of the study protocol and potential risks and answer all the questions raised by patients and their families. Each participant who meets all inclusion criteria and does not meet any exclusion criteria will sign an informed consent form. Upon enrolment, participants will be coded with a unique number.

### Additional consent provisions for collection and use of participant data and biological specimens {26b}

No biological specimens were collected, and we did not use participant data in ancillary studies.

### Interventions

#### Explanation for the choice of comparators {6b}

Mihata proposed SCR with the autogenous fascia latas in 2012 to treat irreparable MRCTs [6]. The SCR can restore the stability of the upper part of the shoulder joint and avoid excessive upwards movement of the humeral head and the impact of the shoulder peak. For irreparable MRCT, SCR can restore the stability of the upper part of the glenohumeral joint and the function of the shoulder joint.

STR involves the fixation of one end of the graft in the footprint and the other end in the medial spine of the scapula, with the aim of achieving muscle-fascial fusion by the 6^th^ postoperative week. Following STR, the SSP may transmit the contraction force to the humerus, thus restoring the anatomy and dynamic function of the rotator cuff. However, whether STR could achieve a better clinical outcome than SCR is still not known.

### Intervention description {11a}

The patient was placed under general anaesthesia in the contralateral decubitus position. Under cuff protection, the upper limb on the affected side was abducted by 30° and flexed at 20°. The vertical traction weight was 3 kg, and the lateral traction weight was 2 kg. The upper limbs on the affected side, neck and shoulders to the midline on the back side, nipple on the affected side of the ventral side, and autogenous fascia latas on the affected thigh were routinely disinfected.

The conventional posterior, anterior and anterolateral portal is selected and used. First, a 30° arthroscope (Smith and Nephew company) was placed into the glenohumeral joint from the posterior portal. The conditions of the subscapularis, labrum, articular cartilage and long head of the biceps are evaluated and repaired through the anterolateral portal. Then, the arthroscope was transferred into the subacromial space from the posterior approach to perform arthroscopic subacromial decompression, and debridement via the IPMRCT was confirmed arthroscopically.

#### STR group

The width of the autogenous fascia latas patch was measured from the anterior to the posterior edge of the rotator cuff tendon lesion. The length of the autogenous fascia latas patch was based on the distance from the lateral edge of the rotator cuff footprint and the distance from the most medial spine of the scapula. The thickness of the autogenous fascia latas was depended on the autogenous fascia latas, however, making sure this graft was 3 to 4 mm in thickness at least. If it is less 3 mm, longer autogenous fascia latas will be harvested and folded. Two absorbable anchors with a diameter of 4.5 mm (GRYPHON for the medial row and VERSALOK for the lateral row; DePuy Mitek Synthes, Raynham, MA) were placed on the medial line of the footprint. The anchors were sutured through the lateral autogenous fascia latas patch, which was 2 cm away from the lateral end, and the traction wire was sutured at the medial end of the autogenous fascia latas patch. The guide pin passed through the subacromial from the lateral portal, ran along the supraspinatus muscle to the most medial part of the spine of the scapula, and penetrated the dorsal skin. The traction wire was pulled medially to pull out the autogenous fascia latas patch under the acromion, and then the wire of the anchor was knotted to fix the autogenous fascia latas graft in a double-row style. A transverse incision of approximately 3 cm in length was made at the most medial side of the scapular spine to expose the medial end of the patch and the spine of the scapula. An absorbable lupine anchor with a diameter of 5.5 mm (Lupine, Depuy Synthes) was implanted at the most medial side of the scapular spine, and the autogenous fascia latas graft was sutured and fixed under the appropriate tension. The incision was sutured layer by layer, and the operation was completed.

#### SCR group

For autogenous fascia latas, the size of the rotator cuff tear and superior capsule were evaluated in both the anteroposterior and mediolateral directions at 45 shoulder abduction by using a measuring probe. The width of the autogenous fascia latas patch was measured from the anterior to the posterior edge of the rotator cuff tendon lesion. The length of the autogenous fascia latas patch was based on the distance from the lateral edge of the rotator cuff footprint and the medial side of the superior glenoid. Autogenous fascia latas was fold for double layers and the thickness was guaranteed to be 6–8 mm. Two threaded anchors were inserted 5–6 mm above the medial side of the superior glenoid according to the surgical condition. Two anchors were inserted behind the midline of the scapula at the 10 to 11 o’clock and 11 to 12 o’clock positions. For the outer anchors, a double-row repair technique was performed on the footprint.

### Criteria for discontinuing or modifying allocated interventions {11b}

1. Intraoperative examination found that the rotator cuff of the subject was “repairable” (if it was determined that the ruptured rotator cuff could be directly repaired during the operation, conventional repair of the ruptured rotator cuff, namely direct repair, would be performed).
2. Deciding to withdraw from the trial at any time and for any reason.
3. Loss to follow-up.

### Strategies to improve adherence to interventions {11c}

All surgical procedures were performed by the same senior doctor and the same 5-person group.

### Relevant concomitant care permitted or prohibited during the trial {11d}

#### Postoperative rehabilitation

Post-surgery, the patients were placed in a brace in a neutral position with a small abduction pillow to protect the reconstructed area. Full active range of motion of the elbow, wrist, and hand was allowed immediately. The patient wore the sling at all times, except while showering and during formal physical therapy, for the first 6 weeks. After 6 weeks, the patient was instructed to perform closed-chain passive table slides and scapular stabilization exercises. Active range of motion and strengthening exercises were started approximately 3 months post surgery.

### Provisions for posttrial care {30}

Trial participants will receive a free MRI scan at 12 months postoperatively and treatment for any complications.

### Outcomes {12}

Imaging data, VAS score, active and passive motion of the shoulder joint, and Constant and AESE scores of the shoulder joint were collected before surgery and 3, 6, 12 and 24 months after surgery. The results of the surgical treatment were studied via preoperative and postoperative imaging scans and various data scores (Table 1).

**Table 1:** Trial schedule according to the recommendation in the interventional trial (SPIRIT) guidelines

Timepoint	Prerandomization	Treatment delivery	3 months postoperative	6 months postoperative	12 months postoperative	24 months postoperative
**Enrolment:**						
Eligibility screen	×					
Informed consent	×					
Baseline questionnaire	×					
**Allocation**						
SCR		×				
STR		×				
**Assessments**						
ASES	×		×	×	×	×
UCLA	×		×	×	×	×
VAS	×		×	×	×	×
Constant-Murley shoulder score	×		×	×	×	×
Range of motion	×		×	×	×	×
Hamada classification	×		×	×	×	×
Shoulder abduction muscle strength (IMS and EMG)	×			×	×	×
Operation time		×				
Intraoperative blood loss		×				

*IMS*, Isokinetic muscle strength testing

#### Primary outcome

The ASES score and the Constant-Murley shoulder score were obtained during the preoperative stage, as well as at 3 months, 6 months, 12 months and 24 months.

#### Secondary outcome

1. Postoperatively, the improvement in shoulder function was evaluated. Additionally, the range of motion was recorded for shoulder joint active forwards flexion, abduction, lateral rotation, and internal rotation behind the back, with the latter recorded using vertebral levels ranging from C1 to S5, classified into grades 1–29. During the preoperative stage, as well as at 3 months, 6 months, 12 months and 24 months postoperatively, the VAS was employed to assess the improvement in shoulder joint pain.
2. The Hamada classification was used to assess the degree of arthritis and measure AHD on anterior–posterior X-ray images during the preoperative stage, as well as at 3 months, 6 months, 12 months and 24 months postoperatively. During the preoperative stage, as well as at 3 months, 6 months, 12 months and 24 months postoperatively, Warner classification and Goutallier grading were employed on MRI oblique sagittal images to evaluate supraspinatus muscle atrophy and fatty infiltration. The integrity of the rotator cuff was determined using the Sugaya classification on MRI coronal images.
3. The operation time and intraoperative blood loss were recorded immediately after the operation, and the postoperative drainage volume was recorded after the operation.

#### Other evaluation

1. Safety indicators: At 6 weeks, 3 months, 6 months, and 12 months following the surgical procedure, the occurrences of adverse events such as infections, fat embolism, deep vein thrombosis in the lower extremities, and retearing were documented.
2. Isokinetic muscle strength testing and surface EMG testing were used to evaluate the improvement in shoulder abduction muscle strength during the preoperative stage, as well as at 6 months, 12 months and 24 months postoperatively.

#### Participant timeline {13}

The participant timeline is shown in Fig. 1.

### Sample size {14}

Sample size calculation for ASES score analysis was performed with an expected standard deviation (SD) of 5.8, a significance level at 5% and power of 0.90 and resulted in 25 participants in each group (50 participants with allowance for 15% drop-out).

In our previous research on postoperative ASES scores of SCR, the score was 87.6 ± 5.8 at the latest follow-up. The best ASES result published by Mihata was 93.1 ± 8.1 (Structural and clinical outcomes after superior capsule reconstruction using an at least 6-mm thick autogenous fascia latas including the intermuscular septum). With a type I error rate of 0.05 (*α* = 0.05, two-tailed) and a power of 90% (*β* = 0.10), the sample size for the study protocol was calculated as 25 patients per group based on the primary outcome indicator by PASS 2021 (Power Analysis and Sample Size, NCSS, LLC, USA). Assuming a 15% drop-out rate, we plan to recruit 58 participants (29 per group) to the study.

### Recruitment {15}

Assuming a 15% drop-out rate, we plan to recruit 58 participants (29 per group) for the study.

## Assignment of interventions: allocation

### Sequence generation {16a}

Randomization will be performed using a computerized randomization procedure in https://castoredc.com. The enrolled patients were randomized in a 1:1 ratio into two treatment groups (the STR group and the SCR group). Patients will be randomized in a 1:1 ratio using variable block sizes of two, four and eight patients, stratified for two treatment groups (the STR group and the SCR group) to avoid imbalances between groups. The order of the block sizes is unknown to the researchers.

### Concealment mechanism {16b}

The random assignment form is usually triplicated. Copies can be enclosed in an opaque envelope, with one copy provided to the patient, one copy to the researcher and one copy to the evaluator. The patient and researchers unseal it before surgery. The allocation scheme is not disclosed to the evaluators collecting outcome data throughout the study. The statistical analysts will also not know whether participants are in the STR or SCR group until the statistical analysis is completed.

### Implementation {16c}

1. This trial requires patients to sign informed consent forms. This study involves private disease information, vital information and so on. The experimental research team will strictly follow the principles of the Declaration of Helsinki and the International Ethical Guidelines for Biomedical Research Involving Humans jointly formulated by WHO and the Council for International Organizations of Medical Sciences to ensure that the subjects’ medical records (medical records/MRI imaging results, etc.) will be kept private. Any public reporting of the results of this study will not disclose the individual identities of the patients.
2. Whether the subjects participate in the study depends entirely on their personal will.
3. Subjects will be asked to withdraw from the study if:
  1. Some test results showed that the subjects were not suitable to continue to participate in the study;
  2. The subjects cannot cooperate with treatment or timely return visits;
  3. During the study period, the subjects developed some new health problems.

## Assignment of interventions: blinding

### Who will be blinded {17a}

In this study, double blinding was used, in which surgeons were aware of the surgical protocol. Evaluators and patients were blinded, to minimize ascertainment bias.

### Procedure for unblinding if needed {17b}

Because a surgical procedure is involved, the surgeons including the principal investigator were unblinded to the trial, and the evaluators collecting outcome data did not join the operation and were blinded throughout the study. When there is serious adverse event reporting and harms or the trial is finished, it will be unblinded.

## Data collection and management

### Plans for assessment and collection of outcomes {18a}

#### Data collection

Shoulder function, pain, imaging, and complications were evaluated at 3, 6, and 12 months after surgery and then every 6 months thereafter.

##### Imaging evaluation

The subacromial space was routinely assessed by radiographs of the shoulder. Shoulder osteoarthritis was assessed according to the HAMADA classification, and the degree of muscle atrophy, fat infiltration and healing of the autogenous fascia latas were evaluated by MRI.

##### Pain score

The relief degree of the patient's shoulder pain was assessed by the VAS scale, where 0 was painless and 10 was severe pain.

##### Evaluation of shoulder function

Constant, UCLA and AESE scores were used to evaluate shoulder recovery and patient satisfaction.

#### Oversight and monitoring

Medical records, registration books, and special records for clinical observation, including doctors’ clinical medical records, are all used in this study.

All data and records relating to clinical observations are kept in the investigator or clinical trial medical facility and made directly accessible to the originator of the clinical trial or the person in charge of the competent authority. Make sure 1 copy of each document and data of the participants, and 1 external HD in the cloud disk of Baidu to guarantee data security.

Clinical observation investigators are responsible for preserving the relevant data collected. No one, other than the members of the study group, ethics committees, or medical regulators, will have access to the data collected in this study without good reason.

#### Quality control

To improve test reliability and reduce differences between operators, the following measures should be taken:

1. Outpatient department by Chief Physician Zhou Binghua;
2. Operations were led by Dr. Zhou Binghua.
3. Photos and video recordings were taken for each operation.
4. One person is responsible for the evaluation and statistical analysis of the test data.

#### Adverse event management

1. Adverse event record
  1. Adverse events: various surgical complications;
  2. Serious adverse events: events that require or prolong hospitalization, cause disability, affect the patient’s working ability, endanger the patient’s life cause death, and lead to congenital malformations during the course of the clinical trial.
2. Adverse event report

Management of adverse events: When adverse events occurred, the investigator should treat them actively and according to surgical management principles without terminating the trial or discontinuing the follow-up. For serious adverse events, in addition to active rescue, the experiment should not be terminated, and observation and follow-up should be continued. The investigator should report to the subject leader within 24 h, timely report to the ethics committee, and record the adverse event in the CRF form. All adverse events should be followed up until remission or stabilization. If the patient dies, a new patient can be enrolled.

### Plans to promote participant retention and complete follow-up {18b}

Study follow-up visits are combined with regular visits at the outpatient clinic to minimize patient burden. The primary outcome is set to be evaluated 3 months after the intervention. All questionnaires can be completed on the day of follow-up.

### Data management {19}

#### Oversight and monitoring

1. Medical records, registration books, and special records for clinical observation, including doctors' clinical medical records, are all used in this study;
2. All data and records relating to clinical observations are kept in the investigator or clinical trial medical facility and made directly accessible to the originator of the clinical trial or the person in charge of the competent authority;
3. Clinical observation investigators are responsible for preserving the relevant data collected. No one, other than members of the study group, ethics committees, or medical regulators, will have access to the data collected in this study without good reason.

### Confidentiality {27}

Clinical observation investigators are responsible for preserving the relevant data collected. No one other than members of the study group, ethics committees, or medical regulators will have access to the data collected in this study without reasonable reason.

### Plans for collection, laboratory evaluation and storage of biological specimens for genetic or molecular analysis in this trial/future use {33}

Not applicable, no samples were collected.

## Statistical methods

### Statistical methods for primary and secondary outcomes {20a}

The statistical software SPSS 25.0 was used for analysis. The normality of metric data, such as ASES scores, Constant scores, VAS scores, shoulder joint mobility, AHD, surgical duration, intraoperative blood loss, and postoperative drainage volume, was assessed using the Shapiro‒Wilk test and graphical analysis. Intergroup differences in metric data conforming to a normal distribution were evaluated using independent samples *t* tests, while nonnormally distributed metric data were assessed using the Mann‒Whitney *U* test. Count variables, such as the occurrence of complications, were evaluated for intergroup differences using the chi-square test. Paired samples *t* test was employed to assess intragroup differences in normally distributed metric data before and after surgery, while nonnormally distributed metric data were represented by median (interquartile range) and assessed using the Wilcoxon rank-sum test. Comparison of ordinal data, such as Hamada classification, Warner classification, Goutallier classification, and Sugaya classification, was conducted using the Wilcoxon rank-sum test. A *p* value less than 0.05 was considered statistically significant.

### Interim analyses {21b}

The interim analysis was performed when 50% of the randomly assigned patients reached the primary endpoint. The interim analysis was carried out by independent statisticians who were blinded to the allocation of treatment. The statisticians will report to the independent Data and Safety Monitoring Commission. The results of the interim analysis will be discussed with the Steering Committee at a joint meeting. The steering committee decided to proceed with the trial and will report to the Central Ethics Committee.

### Methods for additional analyses (e.g. subgroup analyses) {20b}

No additional analyses were performed.

### Methods in analysis to handle protocol nonadherence and any statistical methods to handle missing data {20c}

The loss to follow-up rate will be reduced by adherence to the strategies described above. The sample size is calculated to accommodate a loss to follow-up rate of 15% without affecting statistical power.

### Plans to give access to the full protocol, participant-level data and statistical code {31c}

No longer than 5 years after the collection of the 2-year post-randomization interviews, only the main project principal will deliver a completely deidentified dataset to an appropriate data archive for sharing purposes.

## Oversight and monitoring

### Composition of the coordinating centre and trial steering committee {5d}

The study will be performed by the Sports Medicine Center at Southwestern Hospital. There is no other coordinating centre. The trial steering committee consisted of the ethics committee of the First Affiliated Hospital of Army Medical University (No. AKY2022126) and the clinical research team. The research team consists of two principal investigators who oversee the study and are responsible for medical responsibilities, a study coordinator who plans patient visits, and an investigator who is responsible for data management and trial management. The research team meets weekly to assess progress, identify potential test subjects and work out logistical issues. A statistician was involved in the design of the trial and consulted on statistical issues throughout the study. Study leader Professor Zhou identified the enrolled patients in the outpatient department. A researcher collected general patient information. To improve the reliability of the test and reduce the differences between operators, the following measures were taken: All the operations were led by Dr. Zhou, and all the operations were photographed and recorded by video. The test data were evaluated and counted by one person.

### Composition of the data monitoring committee, its role and reporting structure {21a}

1. Medical records, registration names, and special clinical observation record data, including doctors’ clinical medical records, are used in this study.
2. All data and records relating to clinical observations are kept in the investigator or clinical trial medical facility and made directly accessible to the originator of the clinical trial or the person in charge of the competent authority;
3. Clinical observation investigators are responsible for preserving the relevant data collected. No one other than members of the study group, ethics committees, or medical regulators will have access to the data collected in this study without good reason.

### Adverse event reporting and harms {22}

Management of adverse events: When mild adverse events occur, the investigator should treat them actively and according to surgical management principles without terminating the trial. For severe adverse events, in addition to active rescue, the trial should be terminated and unblind to the rescuer and participants. The observation and follow-up will be continued; however, it will be removed from the study. The investigator should report to the subject leader within 24 h, timely report to the ethics committee, and record the adverse event in the CRF form. All adverse events should be followed up until remission or stabilization. If the patient dies, the patient should be removed from the list of study participants.

### Frequency and plans for auditing trial conduct {23}

The main project principal will be appointed annually to review the quality and compliance of the research for one year. The main project principal will also confirm the integrity of the test. Documents and informed consent and wills were randomly examined for inclusion and exclusion of several study participants.

### Plans for communicating important protocol amendments to relevant parties (e.g. trial participants, ethical committees) {25}

The research results shall be submitted to the Medical Ethics Committee (MEC) in the form of a bulletin. All important protocol amendments will be sent to the MEC for approval again. Upon receipt of the MEC's approval, important protocol amendments will be shared with the participants and implemented. Substantive revisions include, for example, changes to study inclusion and exclusion criteria, study design, interventions, or outcome measures.

### Dissemination plans {31a}

According to the research background, research purpose, experimental design, data sorting results, statistical analysis results and other information, we will write a manuscript and try to publish another paper and make a special report at the academic conference.

## Discussion

Irreparable MRCT presents a special challenge for both patients and surgeons alike. Both STR and SCR were introduced for the repair of irreparable MRCT. In this prospective randomized controlled trial, the application of STR and SCR in the treatment of large unrepairable rotator cuff defects was investigated to provide a more objective approach for the treatment of IPMRCT.

STR could restore the dynamic stability of the shoulder, and SCR could restore the passive biomechanical stability. The superior capsule plays a role as one of the static stabilizers, and superior stability is disrupted because of irreparable MRCT. SCR restored the stability of the glenohumeral joint superiorly at extremes of range of motion [6, 7]. STR and bridging techniques restore dynamic stability by restoring the anatomy and dynamics of the SSP [5]. The difference between STR and bridging techniques was the healing of the interface. In STR, fusion in the medial region is achieved between the graft and the SSP muscle; however, in bridging techniques, healing is achieved between the graft and the SSP tendon. Our unpublished data showed that fascia-muscle fusion is better than fascia tendon healing biomechanically and histologically.

Both STR and SCR achieved a good early clinical outcome. The effectiveness of SCR for repairing IPMRCT was verified [6, 8, 9]. Panzert, J reported that the surgical procedure of STR using open infraspinatus tendon shift and autologous biceps tendon interposition grafts resulted in the successful reconstruction of otherwise nonreconstructable MRCT in 2022 [10]; however, in fact, it was a tendon transfer, not a real STR. Ma and Zhou first reported an anatomical STR using an autogenous fascia latas for irreparable posterosuperior MRCT [5]. STR could transfer the strength of the SSP muscle to the rotator cuff insertion of the greater tubercle of the humerus. The clinical effect of STR is theoretically superior to that of SCR. Generally, STR used 5 anchors compared 6 anchors in SCR and might be less operation time. However, there are no cohort studies of STR or long-term clinical outcomes.

Limitations of this study is not a multi-center study. In addition, arthroscopic reconstruction of the supraspinatus muscle and upper articular capsule of the shoulder requires a learning curve. For surgeons who lack such expertise, surgical effectiveness may be affected.

## Trial status

The study received approval from MEC on October 11, 2022. The current protocol version is V2.3, dated September 5, 2022. Participant recruitment commenced on Jan 1, 2024, with the aim of concluding the recruitment process by Jan 1, 2026. After a 2-year follow-up at least, the research will be finished on Jan 1, 2028.

## Data Availability

This document constitutes the full protocol. Datasets and statistical code used in this study will be available from the First Affiliate Hospital of Army Medical University on reasonable request following the completion of the trial.

## Abbreviations

MRCT: Massive rotator cuff tear
RCT: Rotator cuff tear
SSP: Supraspinatus
IPMRCT: Irreparable posterosuperior massive rotator cuff tear
SCR: Superior capsule reconstruction
STR: Supraspinatus tendon reconstruction
ASES: American Society for Shoulder and Elbow Surgery
ROM: Range of motion
VAS: Visual analogue scale

## References

[1] Mihata T, Lee TQ, Watanabe C, Fukunishi K, Ohue M, Tsujimura T, Kinoshita M (2013). Clinical results of arthroscopic superior capsule reconstruction for irreparable rotator cuff tears. Arthroscopy..

[2] Bi M, Zhou K, Gan K, Ding W, Zhang T, Ding S, Li J (2021). Combining fascia lata autograft bridging repair with artificial ligament internal brace reinforcement: a novel healing-improvement technique for irreparable massive rotator cuff tears. Bone Joint J.

[3] Ulstrup A, Reinhold M, Falster O (2020). Superior capsular reconstruction: 2-year follow-up results. JSES Int..

[4] Claro R, Fonte H (2023). Superior capsular reconstruction: current evidence and limits. EFORT Open Rev.

[5] Ma L, Liao YT, Wang ZY, Li HS, Tang KL, Zhou BH (2023). Supraspinatus tendon reconstruction using fascia lata autograft for irreparable posterosuperior massive rotator cuff tears. Arthrosc Tech.

[6] Mihata T, McGarry MH, Pirolo JM, Kinoshita M, Lee TQ (2012). Superior capsule reconstruction to restore superior stability in irreparable rotator cuff tears: a biomechanical cadaveric study. Am J Sports Med.

[7] Liao YT, Zhou BH, Mihata T (2021). Superior capsule reconstruction: anatomy, biomechanics, indications, and graft treatment. Chin Med J (Engl).

[8] Liao YT, Li HS, Li Y, Tang KL, Li J, Zhou BH (2022). Revascularization character of autologous fascia lata graft following shoulder superior capsule reconstruction by enhanced magnetic resonance imaging. J Orthop Surg Res..

[9] Hasegawa A, Mihata T, Fukunishi K, Itami Y, Uchida A, Neo M (2023). Structural and clinical outcomes after superior capsule reconstruction using an at least 6-mm-thick fascia lata autograft including the intermuscular septum. J Shoulder Elbow Surg.

[10] Panzert J, Hepp P, Hellfritzsch M, Sasse A, Theopold J (2022). Supraspinatus tendon reconstruction using open infraspinatus tendon shift and autologous biceps tendon interposition grafts. Arch Orthop Trauma Surg.

